# INTERGENERATIONAL VIRTUAL YOGA INTERVENTION INVOLVING OLDER ADULTS AND COLLEGE STUDENTS: A PILOT STUDY

**DOI:** 10.1093/geroni/igac059.2231

**Published:** 2022-12-20

**Authors:** Nancy Martens, PhD, Debra Schutte, Thomas Templin, Morgan Cinader-Galvan

**Affiliations:** Wayne State University, Birmingham, Michigan, United States; Wayne State University, Detroit, Michigan, United States; Wayne State University, Detroit, Michigan, United States; Michigan State University, Flint, Michigan, United States

## Abstract

Social isolation is common in older adults and students and is associated with poor health outcomes. While evidence suggests active and yoga interventions improve health, interventions encouraging intergenerational social interaction are limited. The objective was to implement and evaluate a twice-weekly, 6-week, intergenerational yoga intervention delivered virtually. Specific aims included evaluation of feasibility and acceptability, examination of estimates of efficacy on health outcomes, and examination of age cohort effects on acceptability, feasibility, and efficacy. Older adults were recruited through a community activity center and randomized to the control group (N=10) (Mage= 70.05, SD 5.83) or intergenerational older adult treatment group (N=11) (Mage= 72.32, SD= 5.81) with students (N= 12) (Mage= 25.98, SD= 5.64). Older adult outcome measures included Center for Epidemiological Studies Depression Scale (CES-D), PROMIS Social Isolation Scale (SI), Meaning-in-Life Questionnaire, and Tilburg Frailty Index; outcomes for the students included the CES-D, SI, and Image of Aging Scale. Outcomes were assessed at week 0, 3, 6, and 12 (6-weeks post-intervention). Attrition was minimal; 81% of older adults and 80% of students received the full intervention. Using the Acceptability of Intervention Measure, 77.8% of older adults and 100% of college-age adults indicated their willingness to participate again. There were no significant intergenerational effects; however, the effects of yoga were in the expected direction with significant values in CES-D, P < .01 and Meaning-in Life, P < .01. While the acceptability and feasibility of the intervention were supported; additional research is needed to strengthen the intergenerational component of the intervention.

